# Psychological distance and user engagement in online exhibitions: Visualization of moiré patterns based on electroencephalography signals

**DOI:** 10.3389/fpsyg.2022.954803

**Published:** 2022-09-15

**Authors:** Jingjing Li, Ye Yang, Zhexin Zhang, Nozomu Yoshida, Vargas Meza Xanat, Yoichi Ochiai

**Affiliations:** ^1^Graduate School of Comprehensive Human Sciences, University of Tsukuba, Tsukuba, Japan; ^2^R&D Center for Digital Nature, University of Tsukuba, Tsukuba, Japan; ^3^College of Design and Innovation, Tongji University, Shanghai, China; ^4^Faculty of Library, Information and Media Science, University of Tsukuba, Tsukuba, Japan

**Keywords:** COVID-19, online exhibition, psychological distance, electroencephalography (EEG), user engagement (UE), user experience (UX), moiré patterns

## Abstract

The COVID-19 pandemic has significantly affected the exhibition of artworks in museums and galleries. Many have displayed their collection online. In this context, experiencing an online exhibition is essential for visitors to appreciate and understand the artwork. Compared with offline exhibitions, visitors to online exhibitions are often unable to communicate their experiences with other visitors. Therefore, in this study, by facilitating communication *via* Zoom call, we established a system that allows two people to visit the museum together through the Google Arts and Culture (GA&C) website. To reduce the psychological distance between online visitors and help increase user engagement, we designed and developed a media device based on moiré pattern visualization of electroencephalography (EEG) signals. The participants were divided into two groups to visit the online museum, communicating remotely through Zoom. The objective of this study was to test whether a real-time EEG signal visualization device could help close the psychological distance between participants and whether it could increase user engagement with the online exhibition. Participants were randomly assigned to either the normal online exhibition experience (NOEE) group or EEG signal visualization device (ESVD) group. Participants in the NOEE group experienced four online exhibitions (Task1, Task2, Task3, and Task4) together (two participants per test unit). The conditions for participants in the ESVD group remained the same, apart from adding a media device to enable them to visualize EEG signals. A total of 40 university students participated in this study. Independent samples *t*-tests revealed that participants in the ESVD group perceived a significantly closer psychological distance between themselves and the participants on the opposite side than those in the NOEE group (*t* = −2.699; *p* = 0.008 < 0.05). A one-way ANOVA revealed that participants experienced Task3 with significantly closer psychological distance assessments than Task1 (*p* = 0.002 < 0.05), Task2 (*p* = 0.000 < 0.05), and Task4 (*p* = 0.001 < 0.05). Repeated ANOVAs revealed that participants in the ESVD group had higher overall user engagement than those in the NOEE group, with marginal significance (*p* = 0.056 < 0.1). Thus, this study shows that EEG visualization media devices can reduce the psychological distance between two participants when experiencing an online exhibition. Moreover, it can increase user engagement to some extent.

## Introduction

COVID-19 has impacted many areas of our daily lives, including the exhibition of artworks. Most museums and galleries have restricted the number of visitors to prevent the spread of COVID-19 by ensuring social distancing. In public health, social distancing is also called physical distancing (Hensley, 2020; Venske, 2020). Physical distancing involves staying at least 6 ft away from others to avoid contracting an airborne disease such as COVID-19. It is a set of non-pharmaceutical interventions or measures designed to prevent the spread of infectious diseases by maintaining physical distance among people and reducing the number of times people come into close contact (Perra, 2021). Physical distance maps the psychological distance between individuals, reflecting the degree of intimacy in interpersonal relationships (Shenghua, 1997, pp. 118–119). Physical distance is even more important for museum and art gallery visitors, especially in terms of processing, experiencing, and appreciating art. Therefore, in pandemic situations, visitors are limited in viewing the exhibition, since it is difficult to convey ideas to others and listen to their thoughts and feelings.

In 1912, Bullough developed the concept of psychological distance in the aesthetics field to illustrate those esthetic feelings stem from the psychological distance that an observer perceives between themselves and the artwork (Dhar and Wertenbroch, 2000; Chen and Li, 2018). In light of this, in the pandemic and post-pandemic era, the categories of distances in Personal Space Theory proposed by Hall (1959) were diminished, losing the “intimate distance” (0–0.45 m, 0–1.5 ft), “personal distance” (0.45–1.2 m, 1.5–4 ft), and part of the “common distance” (1.2–3.6 m, 4–12 ft) and “public distance” (3.6–7.6 m, 12–25 ft). Hence, the distances of Personal Space Theory have changed. On the other hand, and more importantly, online communication is becoming more common; for example, online classes (Pokhrel and Chhetri, 2021), online work (Blanchard, 2021), and online psychotherapy (Weinberg, 2020); this phenomenon has accelerated online communication and interactions and even become a necessity for many.

As a result of COVID-19, the trend of online communication has become more prominent; consequently, many museums and art galleries have provided online viewing experiences to visitors. International Council of Museums (ICOM) reported that online activities would continue to increase, particularly by creating new digital communication channels in the wake of lockdowns. Additionally, an increasing number of museums and galleries are planning to add online exhibitions to their activities after the end of lockdowns (an increase of 5.6%), and more are planning to start online exhibitions (an increase of 6.5%; International Council of Museums, 2021, pp. 15–16). Digital communication activities increased by at least 15% in museums in Europe and Asia, while the Network of European Museum Organisations (2020) reported that 58% of museums held digital activities (an increase of 37%) and 23% started new activities (King et al., 2021). Thus, the trend of online exhibitions is in line with the trend of online activities. Additionally, online exhibitions are more inclusive, allowing access to people who normally do not have access to physical museums and galleries.

The Internet network makes it possible to chat across time and space and seems to make the distance between people disappear. However, something inherently embedded in the virtual world distorts communication when using AI-facilitated chatting tools (Radziwill and Benton, 2017). Overall, we face uncertain and confused distances both in the physical and virtual worlds (Dewey, 2018), bringing us into a new era of a “distance crisis.” Psychologically distant objects and events are not present in a direct experience of reality. In this case, distance is not limited to the physical surroundings, and it could also be abstract (Trope and Liberman, 2010).

With the development of online exhibition technologies, digital devices, such as virtual reality (VR), 3D panoramic VR, and 3D web engines have been used to display artwork (Styliani et al., 2009). Cell phones, computers, tablets, and interactive multimedia devices [e.g., VR/augmented reality (AR) devices], as information dissemination media and bearers of digital technology (Chang et al., 2014; Wu and Li, 2022), gradually change people’s exhibition viewing modes, making their experiences more human and emotional. The Google Arts and Culture (GA&C) website is one of the most representative online exhibition sites, featuring selected content from more than 2000 top museums, galleries, and archives (Google Arts and Culture, 2018). Therefore, we selected four different interactive exhibitions from the GA&C website as experiential tasks in our experiment. As visiting and appreciating artworks is also a process of mutual communication and exchange of feelings among visitors, online exhibitions are also increasingly enabling online multiplayer experiences to increase interaction and communication between users. For example, the GA&C website features an interactive multiplayer game called “Puzzle Party” in which participants work together to put together scattered pieces of the puzzle based on reference images of the artwork (Google Arts and Culture, 2020). However, only a few studies have focused on exploring the psychological distance between users in remote interactions.

Our study focuses on exploring a new way of interaction to decrease the psychological distance between participants in online exhibitions. Because people’s physiological signal data are closely related to their emotional and physical states and reactions, we aim to express the “distances” between people by visualizing physiological signals. Among physiological signals, brainwave signals originate from voltage fluctuations caused by ionic currents within brain neurons and have been shown to represent macroscopic activities on the surface layers of the brain (Niedermeyer and da Silva, 2005). Considering this, our study aimed to apply electroencephalography (EEG) signals to explore new physical distance expressions. Currently, an increasing number of studies are incorporating user EEG signals. Relevant studies and applications exist in many fields, such as user experience analysis during human–computer interaction (Lee, 2004; Frey et al., 2013; Li et al., 2022b), driver fatigue detection (Jap et al., 2009; Borghini et al., 2014; Gao et al., 2019), emotion recognition (Alarcao and Fonseca, 2017; Shu et al., 2018), etc. Additionally, more studies have used headset-based noninvasive brain–computer interfaces (Cincotti et al., 2008; Cecotti, 2011). These studies demonstrated that EEG headset devices have increased accuracy (Maskeliunas et al., 2016; LaRocco et al., 2020; Fu et al., 2022).

This novel form of distance expression needs to be visualized to help people’s awareness of “distance.” The data visualization of various types of information has always been an effective way to transform abstract data and concepts into clearly understandable images. Many fields have applied EEG signal visualization, including affective visualization (Liu et al., 2010; Fu and Li, 2022), interactive art exhibitions (Christopher et al., 2013, 2014), gaming experiences (Kerous et al., 2018), etc., to enhance participants’ understanding and visual perception of their EEG signal changes. Therefore, distance visualization can enhance the perception of human interaction.

We simulated two participants together experiencing four online exhibitions from the GA&C website by designing a system. This system was as follows: the two participants were in the same room, and a computer and a screen displaying the EEG signal visualization was assigned to each participant. As the participants were separated by two screens, they could not see each other, thus simulating the scenario of real remote interaction. The experiment used in this study was a comparison experiment. The only independent variable was a media device that transformed the difference in EEG signals between the two participants into real-time moiré patterns. From the 17th to the 20th century, moiré patterns were gradually discovered and explored in mathematics, physics, and art (Isaac, 2000). These were defined as interference images produced by more than two similar fence-like overlapping stripes. Participants were divided into two groups, one of which communicated through Zoom with the camera turned on during the exhibition. The other group was exposed to EEG signal visualization screens. Each screen was placed in front of the participant. During the intervention, participants were able to view the changes in the moiré images displayed on the screens.

Our research questions are as follows:

- R.Q.1: Does visualization of EEG signal differences in the online exhibition experience help users reduce psychological distance?
- R.Q.2: Can EEG enhances user engagement in online environments?

## Materials and equipment

### Participants

We considered a sample of university students. They were deemed suitable for testing whether the addition of an EEG visualization device would help improve psychological distance and user engagement, given the operational complexity of online exhibitions and evaluation of engagement in online experiences. Before we recruited the participants, we designed our experiments in accordance with the Declaration of Helsinki by the World Medical Association. The ethics review office of the Faculty of Library, Information and Media Science of the University of Tsukuba in Japan approved the study (permission number 22–4).

In the notice for recruiting participants, we marked the general content and flow of the experiment, explicitly stating that two people were required to participate in the experiment as a unit. To reduce the effect of the difference in familiarity between the two participants in each experiment on psychological distance, we selected participants who had known each other for a long time (65% of participants were mutually acquainted for more than 1 year) and were mainly from the same research laboratory. Before starting the experiment, we informed the participants that there was no compensation for participation in the experiment. After the participants received a complete explanation of the study, they agreed to participate and subsequently signed a written informed consent form. Permission of publication of any potentially identifiable images or data included in this study was obtained from pertinent individuals.

### Materials

#### Experimental scenarios and equipment

The experiment location was a classroom with an area of 39 m^2^ equipped with Wi-Fi. The equipment used during the experiment included the following: six single light wood-colored desks, made of particleboard covered with a laminate; two Mac laptops displaying the online exhibition; two Apple earphones; two EPOC X headsets for capturing EEG signals; two laptops for recording EEG signal data; two 70-in screens displaying moiré patterns based on EEG signals; two iPads for answering questionnaires; and one DJI Pocket 2 video camera for recording the whole experiment.

#### Google arts and culture

In this study, we classified online exhibition websites into four types based on the differences in the interaction methods between exhibits and users on the GA&C website. The GA&C Project was launched in 2011 in collaboration with 17 collaborating museums. The original 1,061 high-resolution images (created by 486 artists from different backgrounds) were displayed in 385 virtual exhibition rooms with 6,000 street-view-style panoramic images (AtoZ Wiki, 2011; Kennicott, 2011). With the aim of making culture more accessible, the project digitized millions of artifacts and made them available online, accessible to everyone (Kennicott, 2011).

Google Arts and Culture has a wealth of content and features, including Virtual Museum Tour, Explore and Discover, Zoom Views, Create Your Own Collections, and educational content. The homepage of the GA&C website is divided into different modules based on these contents and functions, including 2D images and information, 3D virtual space, game interaction, and video explanation. Based on the functions and modules mentioned above, we selected four corresponding exhibitions based on four different interaction types on the GA&C website as tasks in this experiment. The four interaction types are as follows:

- Task1: 2D information kiosk; this describes to the visitor what the exhibit expresses, specifically narrative logic, through pictures/text/diagrams, etc.
- Task2: 3D virtual exhibition; this is a virtual recreation of physical three-dimensional (3D) exhibitions or museums that allow a visitor to navigate in a way that is closer to reality.
- Task3: Interactive game; the user can complete the game (puzzles/coloring games/photography games) tasks with artworks in single or multiplayer mode.
- Task4: Video instruction; this explains information related to the artwork through dynamic video (including motion graphics and sound effects).

#### The EEG signal visualization device

The human brain contains neurons that communicate *via* electrical impulses. EEG signal measurement is a practical method for detecting sequential changes in brain activity without significant time delays. When we attempted to communicate the relationships between the two brainwave images, we automatically associated them with moiré patterns. Brainwaves and moiré patterns have many common characteristics, not only in principles but also in visual properties.

Illusory patterns always appear as water ripples when shooting a screen with digital devices. This type of pattern is called moiré, which is accidental, transient, and fluid; while it is easy to ignore, it contains variable visual forms (Spillmann, 1993).

**Figure 1:**
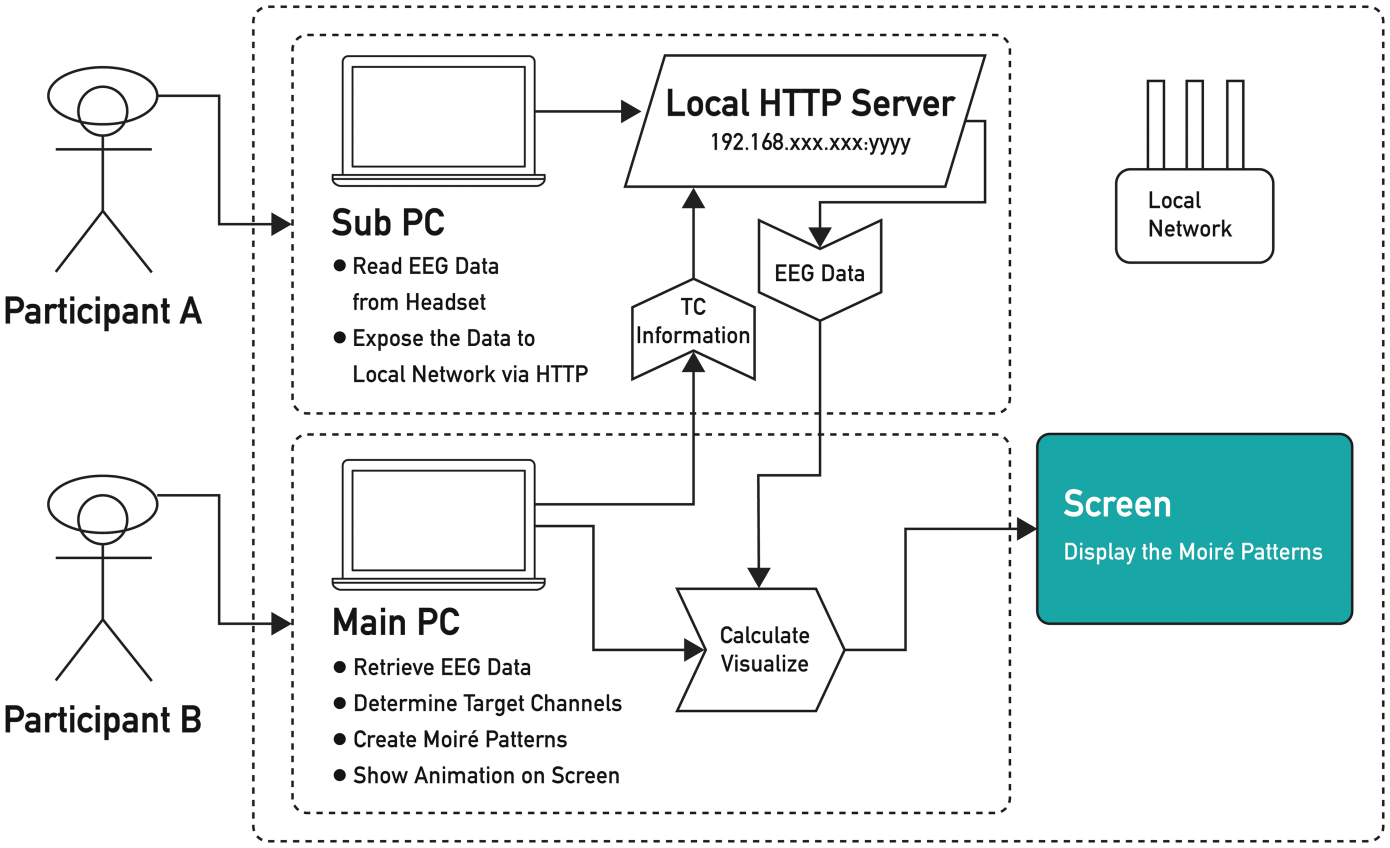
Flowchart of the operation of the experimental platform.

**Figure 2:**
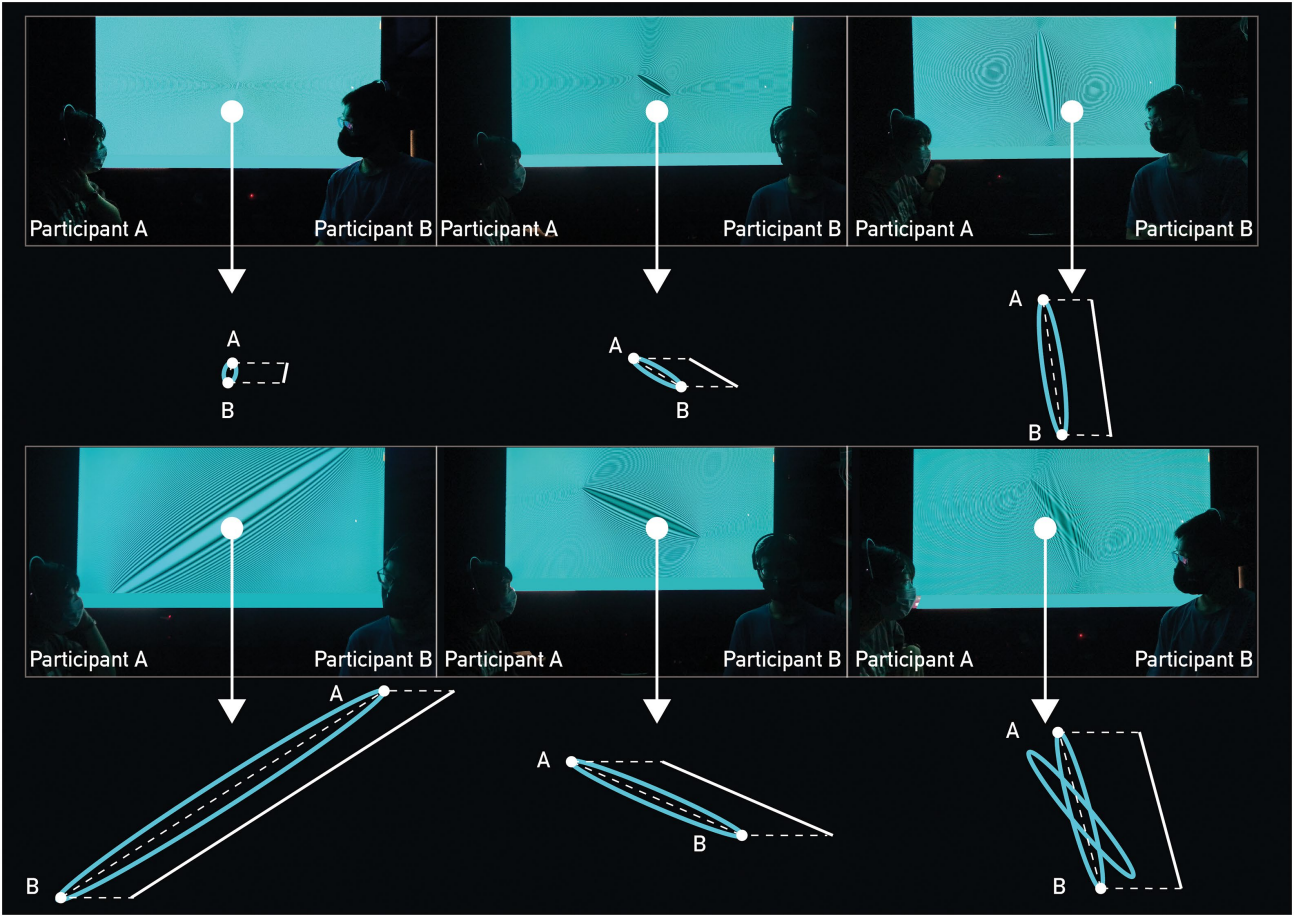
We transformed the difference between the electroencephalography (EEG) signals of participant A and participant B into the moiré pattern, and the pattern displayed six different states on the monitor (the images of the six states are six randomly selected time moments).

We conducted two experiments (Li et al., 2022a). The operating principle of the EEG signal visualization device is to detect the participants’ EEG signals; therefore, we built a platform to calculate the differences in real time (see Figure 1). We applied moiré patterns to visualize EEG signal discrepancies to create the following analogy: when the brainwaves from two participants (A and B) were in phase and got more similar, the generated moiré patterns consequently had smaller sizes with shorter diameters, echoing constructive interferences and shorter “distances” between the two participants, and vice versa (see Figure 2). The six scenario images in Figure 2 represent images on the monitor at six different time points. The distance between the two endpoints of the graph on the screen indicated the numerical difference between participant A and participant B’s EEG signals and real-time changes. Therefore, when the difference between the EEG signals of the two participants decreased, the shape of the moiré pattern became smaller.

#### Questionnaire 1: Psychological distance mapping diagram

**Figure 3:**
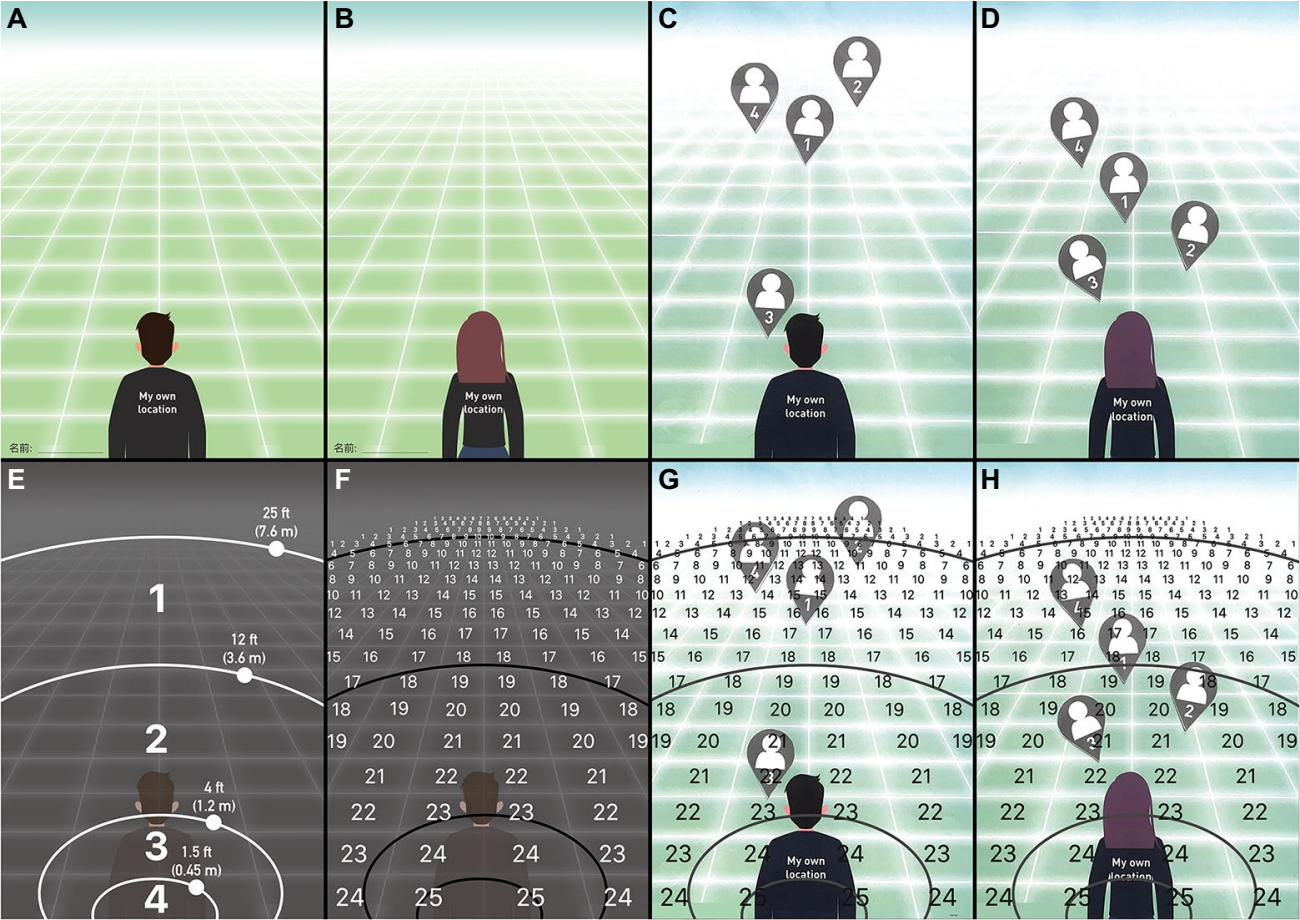
**(A)** Visual stimulus material for those who identified as males; **(B)** Visual stimulus material for those who identified as females; **(C)** Example of a worksheet completed with four tasks of the humanoid card, pasted by a participant who identified as male; **(D)** Example of a worksheet completed with four tasks of the humanoid card, pasted by a participant who identified as female; **(E)** Classification of personal space represented in visually stimulating materials; **(F)** Specific scores (from 1 to 25 scores) for each square represented in the visual stimulus material; **(G)** Example of a worksheet (completed by a participant who identified as male) that displayed scores when counting scores; and **(H)** Example of a worksheet (completed by a participant who identified as female) that displayed scores when counting scores.

We designed a picture of a male or female (non-binary gender people could independently choose a closer image) to help participants enhance their identification with the questionnaire (see Figures 3A,B). The concepts of distance and location were evoked by a paper questionnaire printed with an image that suggested the participants’ positional orientation and 3D space (Kundrát and Rojková, 2021). The image was printed on A4 paper, and the participants achieved placement by pasting pre-prepared cards (corresponding to the four tasks). The participants used different prompts to identify which part of the image was closer or farther away. One of the cues was a linear perspective convergence grid supported by the texture gradient coverage.

The participants positioned the given cards on the pictures (the cards represented images of participants interacting together) according to the evoked notions of proximity and distance. After each completed online exhibition experience, they received neutral oral instructions to “place this card on the picture depending on whether you feel close or far from the object of interaction” (see Figures 3C,D).

According to the distance theory, we divided the picture into four zones (Hall, 1959): intimate distance (0–0.45 m, 0–1.5 ft), personal distance (0.45–1.2 m, 1.5–4 ft), social distance (1.2–3.6 m, 4–12 ft), and public distance (3.6–7.6 m, 12–25 ft; see Figure 3E). Additionally, we arranged the corresponding score for each grid separately, from 1 to 25 points (see Figure 3F). The closer the participant, the higher the score value, and vice versa (see Figures 3G,H).

#### Questionnaire 2: User engagement scale-short form

The User Engagement Scale-SF (UES-SF) is a measure developed to evaluate user engagement and has been used in a variety of digital applications (O’brien and Toms, 2013). It is designed to measure six attributes of user engagement: aesthetic appeal, focused attention, novelty, perceived usability, sensory engagement, and persistence. The UES-SF is a short form (SF) of the User Engagement Scale (UES), shortened from 31 to 12 items, reducing the response time burden on users (O’Brien et al., 2018). It consists of the following components.

- FA: Focused attention, including three items.
- PU: Perceived usability, including three items.
- AE: Aesthetic appeal, including three items.
- RW: Reward factor, a set of elements (three elements) consisting of EN (persistence), NO (novelty), and FI (sensory involvement) components of the User Engagement Scale.

### Design

**Figure 4:**
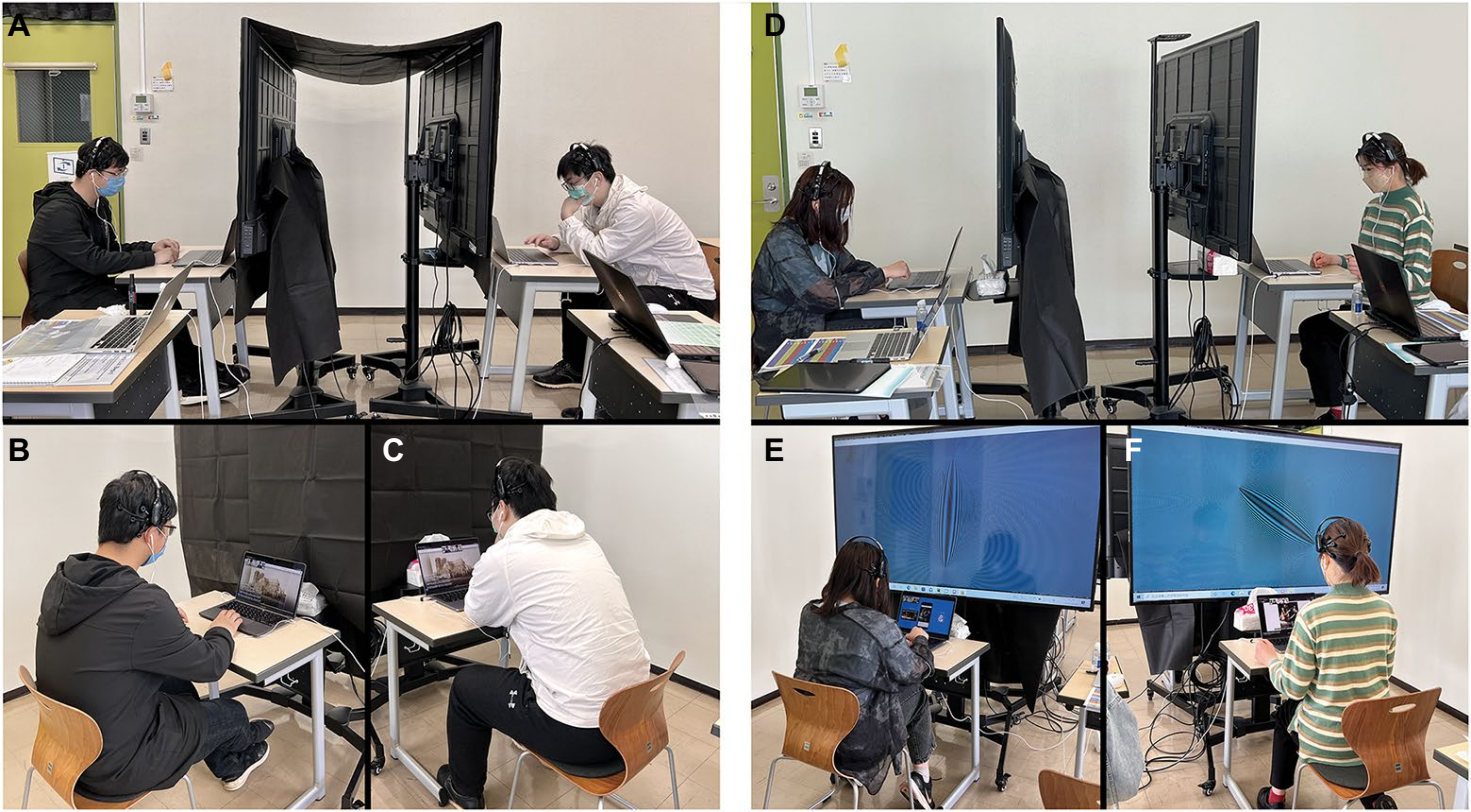
**(A)** The normal online exhibition experience (NOEE) group’s overall experimental scene; **(B)** The experimental scene of a participant in the NOEE group; **(C)** The experimental scene of another participant in the NOEE group; **(D)** The EEG signal visualization device (ESVD) group’s overall experimental scene; **(E)** The experimental scene of a participant in the ESVD group; and **(F)** The experimental scene of another participant in the ESVD group.

In the between-subjects design, we designed two conditions, and each participant was tested in only one of the conditions (see Figure 4). The participants were randomly assigned to two groups. These two groups were based on two conditions: the normal online exhibition experience group (NOEE group) and the group with an additional EEG signal visualization device (ESVD group). The difference between the two groups is that we added an independent variable, a media device that translates the difference in EEG signals between two participants into real-time moiré patterns, in the ESVD group. The total duration of the experiment was 5 days (10:00–18:00/day), with the NOEE group performing on Day 1–2.5 and the ESVD group on Day 2.5–5.

### Procedures

At the beginning of the experiment, the implementer explained the procedure to the participants and asked them to fill in their personal information. To capture the EEG signals in real time, the participants wore the EPOC X headset-based EEG equipment. Simultaneously, the experiment implementer used two other computers to record the captured data using EMOTIVPRO 3.0.

**Figure 5:**
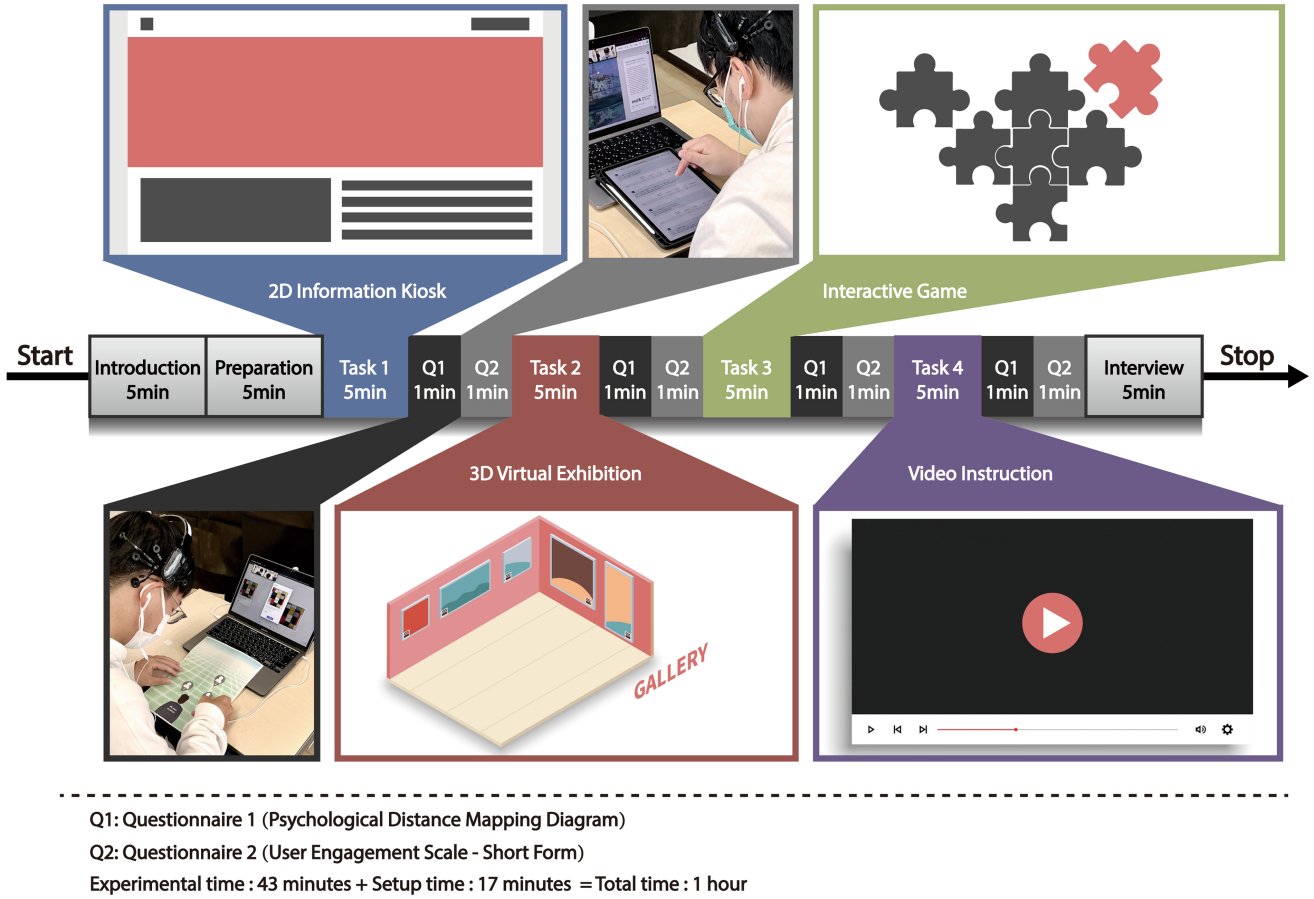
Flow diagram of the experimental procedures.

Subsequently, the participants moved on to the next task until all four tasks and questionnaires were completed within 1 h (see Figure 5). The participants learned how to operate Tasks 1–4 in online exhibitions to familiarize themselves with the specific interaction processes and used the PC’s touch area to control the interface. The PC’s built-*in camera* and Zoom were used to display images of the faces of the participants in real time during the experiment. Each PC was connected to an earphone that was used by the participants for communication. After visiting each exhibition, participants were required to answer a paper questionnaire (PDMD) and an electronic questionnaire (UES-SF) on an iPad.

In contrast to the NOEE group, in the ESVD group, the experimental implementer explained the EEG visualization media device to the participants before the start of the experiment. Additionally, this media device was used as a background screen (the background in front of both participants showed the same view) during the online museum experience, which participants could freely view at any time.

At the end of the experiment, we asked participants to write their feedback based on their overall experience of the experiment by describing the response requirements in the relevant field in the questionnaire. The headings in the questionnaire were described in three languages: English, Japanese, and Chinese, and the participants responded in their native language. The experiment implementer assisted the participants in this process by explaining the questionnaire titles by using neutral vocabulary and did not guide the content of the participants’ responses.

### Statistical analysis

We used SASS version 25.0 for statistical analysis of the data collected during the experiment. We used descriptive statistics for the sample and divided the experimental data into three main parts:

- Questionnaire data on the characteristics of the experimental participants, including gender, duration of mutual acquaintance, and most frequently used software for online meetings.
- Questionnaire data on the perceived psychological distance of the participants during their experience with Tasks 1–4.
- Questionnaire data on the user engagement of the participants during their experience with Tasks 1–4.

We utilized the following analysis methods:

- Experimental participants: We analyzed the differences in age between groups using independent samples *t*-tests. Differences in gender, region, duration of mutual acquaintance with the other participants, and the most frequently used online meeting software were also analyzed using *χ*^2^ tests with SASS version 25.0.
- To solve R.Q.1: Regarding psychological distance during the interactions, we used one-way ANOVA and an independent samples *t*-test to investigate the differences among the four tasks within groups and the users between the groups.
- To solve R.Q.2: Finally, UES-SF questionnaires were analyzed with repeated ANOVA to determine the differences in user participation between groups.

## Results

### Feasibility and participation

A total sample of 40 students from the University of Tsukuba participated in this study, including 20 males, 19 females, and one non-binary gender person. All participants were East Asian (specifically, Japanese and Chinese). The NOEE group included 20 participants (12 males and eight females) with a mean age of 24.9 ± 1.5 years. In the NOEE group, 14 participants were mutually acquainted for more than 1 year for two people involved in the same experiment (additionally, two participants were mutually acquainted for more than 6 months less than 1 year, two participants for more than 1 month less than 6 months, and two for less than 1 month). The NOEE group included 12 participants who used Zoom most frequently for online meetings, and eight participants used Microsoft Teams. The ESVD group consisted of 20 participants (eight males, 11 female, and one non-binary gender individual) with a mean age of 24.3 ± 2.3 years. In the ESVD group, 12 participants (comprising the groups of two people involved in the same experiment) were mutually acquainted for more than 1 year (in addition, four participants were mutually acquainted for more than 6 months and less than 1 year, two participants for more than 1 month and less than 6 months, and two participants for less than 1 month). The ESVD group had 15 participants; they used Zoom most frequently for online meetings, and five participants used Microsoft Teams. There were no significant differences between the groups in terms of mean age (*p* = 0.375), sex ratio (*p* = 0.343), duration of acquaintance ratio (*p* = 0.946), and commonly used online meeting software proportion (*p* = 0.501). Further details are provided in Table 1.

**Table 1:** Participant characteristics.

Characteristics	NOEE Group (***N*** = 20)	ESVD Group (***N*** = 20)	Statistics	
			T or χ^2^	*p*
**Age mean (SD)**	24.9 (1.5)	24.3 (2.3)	*T* = 0.897	0.375
	***N*** **(%)**	***N*** **(%)**		
**Gender**				
Male	12 (60)	8 (40)	χ^2^ = 2.191	0.343
Female	8 (40)	11 (55)		
Non-binary gender	0 (0)	1 (5)		
**Duration of acquaintance**				
>1 year	14 (70)	12 (60)	χ^2^ = 1.061	0.946
0.5 years to 1 year	2 (10)	4 (20)		
1 month to 0.5 years	2 (10)	2 (10)		
1 month>	2 (10)	2 (10)		
**Online meeting software**				
Zoom	12 (60)	15 (75)	χ^2^ = 1.026	0.501
Teams	8 (40)	5 (25)		

NOEE group, online exhibition experience group; ESVD group, additional EEG signal visualization device group.

All participants in the NOEE and ESVD groups completed the appropriate experimental processes without encountering any technical difficulties or significant participant disturbances, which might have terminated the experience. We observed the participants’ behavior and determined that all participants were focused on and motivated from the beginning to the completion of the trial.

### Evaluation of psychological distance

We divided the results of the participants’ psychological distance assessment into three parts:

- Whether there was a difference between the four tasks within the NOEE group
- Whether there was a difference between the four tasks within the ESVD group
- Whether there was a difference between the NOEE and ESVD groups

In the NOEE group, there were statistical differences among the four tasks within the group according to the results of the one-way ANOVA (*F* = 7.473; *p* = 0.000; see Table 2). According to the results of multiple comparisons, the scores of Task3 were higher than those of the other three tasks, and there were significant differences between the scores of Task3 and Task1 (*p* = 0.002), Task2 (*p* = 0.000), and Task4 (*p* = 0.001; Table 3).

**Table 2:** One-way ANOVA within the NOEE group.

**Task**	***N***	**Mean ± SD**	***F***	***p***
Task1	20	17 ± 4.46	7.473	0.000^*^
Task2	20	16.35 ± 4.146		
Task3	20	21.45 ± 1.877		
Task4	20	16.7 ± 4.578		

* Significance at the 0.05 level.

**Table 3:** Multiple comparisons between Task1/2/3/4 within the NOEE group.

**(I) Task**	**(J) Task**	**MD (I-J)**	***SD***	***p***
Task1	Task2	0.65	1.362	0.997
Task1	Task3	−4.45	1.082	0.002^*^
Task1	Task4	0.3	1.429	1
Task2	Task3	−5.1	1.018	0^*^
Task2	Task4	−0.35	1.381	1
Task3	Task4	4.75	1.106	0.001^*^

* Significance at the 0.05 level.

In the ESVD group, there were significant differences between the four tasks within the group according to the results of the one-way ANOVA (*F* = 4.510, *p* = 0.006; see Table 4). According to the results of multiple comparisons, the scores of Task3 were higher than those of the other three tasks, and there were significant differences between Task3 and Task1 (*p* = 0.048), Task2 (*p* = 0.001), and Task4 (*p* = 0.021; Table 5).

**Table 4:** One-way ANOVA within the ESVD group.

**Task**	***N***	**Mean ± *SD***	***F***	***p***
Task1	20	19.2 ± 3.694	4.510	0.006^*^
Task2	20	18.55 ± 2.964		
Task3	20	21.85 ± 1.981		
Task4	20	18.65 ± 4.043		

* Significance at the 0.05 level.

**Table 5:** Multiple comparisons between Task1/2/3/4 within the ESVD group.

**(I) Task**	**(J) Task**	**MD (I-J)**	***SD***	***p***
Task1	Task2	0.65	1.059	0.989
Task1	Task3	−2.65	0.937	0.048^*^
Task1	Task4	0.55	1.224	0.998
Task2	Task3	−3.3	0.797	0.001^*^
Task2	Task4	−0.1	1.121	1
Task3	Task4	3.2	1.007	0.021^*^

* Significance at the 0.05 level.

According to the results of statistical tests (independent samples *t*-test) between the four tasks in the NOEE and ESVD groups, there were no significant differences between Task1 in the NOEE group and Task1 in the ESVD group (*p* = 0.098), between Task2 in the NOEE group and Task2 in the ESVD group (*p* = 0.061), between Task3 in the NOEE group and Task3 in the ESVD group (*p* = 0.516), and between Task4 in the NOEE group and Task4 in the ESVD group (*p* = 0.162). After analyzing the two groups’ overall engagement (OE), the mean ± SD was 17.88 ± 4.379 for the NOEE group and 19.56 ± 3.478 for the ESVD group. Furthermore, a statistical test (independent samples *t*-test) for these two groups yielded T = −2.699, *p* = 0.008, and *p* < 0.05. This result indicated a statistical difference between the NOEE and ESVD groups (Table 6), where the ESVD group had higher scores than the NOEE group.

**Table 6:** Independent sample *t*-test for Task1/2/3/4 and overall Task between NOEE and ESVD groups.

**Task and OT**	**NOEE Group Mean ± SD**	**ESVD Group Mean ± SD**	**T**	**p**
Task1	17 ± 4.46	19.2 ± 3.694	−1.699	0.098
Task2	16.35 ± 4.146	18.55 ± 2.964	−1.931	0.061
Task3	21.45 ± 1.877	21.85 ± 1.981	−0.656	0.516
Task4	16.7 ± 4.578	18.65 ± 4.043	−1.428	0.162
OT	17.88 ± 4.379	19.56 ± 3.478	−2.699	0.008^*^

NOEE group, online exhibition experience group; ESVD group, additional EEG signal visualization device group; OT, overall task.

* Significance at the 0.05 level.

### Evaluation of user engagement

We evaluated user engagement in the NOEE and ESVD groups, first by detecting within-subject effects for different indicators in the two groups, followed by comparing the two groups in pairs.

Table 7 illustrates the repeated-measures ANOVA for all participants’ scores to compare the differences between the four tasks. The four tasks differed significantly in the PU indicator (*F* = 4.559, *p* = 0.034), but not in the FA (*F* = 0.504, *p* = 0.479), AE (*F* = 1.968, *p* = 0.163), and RW (*F* = 2.587, *p* = 0.11). Table 8 presents the paired comparisons of the overall engagement values for the two groups (NOEE and ESVD). Paired comparisons showed that participants’ overall engagement was marginally significantly higher in the ESVD group than in the NOEE group (*p* = 0.056; 0.05 < *p* < 0.01).

**Table 7:** Within-subjects effect test for different indicators under the NOEE and ESVD groups.

**UES-SF**	**SS**	**df**	**MS**	***F***	***p***
Focused attention (FA)	1.008	1	1.008	0.504	0.479
Perceived usability (PU)	9.633	1	9.633	4.559	0.034^*^
Aesthetic appeal (AE)	3.169	1	3.169	1.968	0.163
Reward factor (RW)	3.502	1	3.502	2.587	0.11
Overall engagement (OE)	3.763	1	3.763	3.715	0.056

* Significance at the 0.05 level.

**Table 8:** Paired comparisons for the NOEE and ESVD groups.

**(I) Group**	**(J) Group**	**MD (I-J)**	***SD***	***p***
NOEE Group	ESVD Group	0.177	0.092	0.056

## Discussion

The experimental results showed that increasing the visualization of participants’ EEG signals during the GA&C website experience could reduce perceived psychological distance to some extent. In comparing the analysis of user engagement on the GA&C website, we found that the ESVD group, that is, the group with added the EEG signal visualization media, had a slightly higher user engagement. Although the psychological distance is a widely used theoretical construct in literature, there is little clarity on what makes things appear to have greater or less psychological distance. Our study found that EEG signal visualization reduces the perceived psychological distance to some extent and enhances user engagement.

### Social distance and technology

There are various ways of experiencing online exhibitions, including cell phones, tablets, VR devices, and computer screen displays (Broeck et al., 2017). Different experience methods have different degrees of impact on users’ psychological distance. During the COVID-19 pandemic, emerging technologies such as tracking apps, AI, big data, 5G, drones, and robotics are being used in China and Japan (Shaw et al., 2020). The high penetration rate of the Internet and smartphones, combined with the acceptance of new technologies, particularly among urban youth in these countries, may have contributed to the positive perception of the moiré patterns generated in this study.

By comparing the four exhibitions within the group, we found that different interaction methods had other effects on the psychological distance between the users. In Task3, two participants were required to work together to complete a pair of puzzles. In this task, compared with the other tasks, they could see the trajectory of each other’s operations in real time, and the displayed operation interface was synchronized. The synchronized interface provided an additional way for participants to understand each other’s emotions. The statistical results showed that in the NOEE and ESVD groups, the users’ psychological distance was the closest in the experience of Task3 (interactive puzzle game) than the others (see the section “Evaluation of Psychological Distance,” for a description of the within-group comparison results). Additionally, the participants had the highest number of conversations during the interaction of Task3, and more than half of them expressed a desire to continue experiencing Task3.

Synchronization is considered a joint change in psychophysiological signals when people interact intensively with each other (based on Ekman et al., 2012). Such phenomena while playing games have proven to be psychologically and physically beneficial (e.g., Motataianu, 2015; Robinson et al., 2020). Synchronization can increase socialization capabilities through shared interactions and online experiences (e.g., Sadhukhan et al., 2021). Given that art as a shared experience can also foster emotional contagion in viewers (Gernot et al., 2018), combining online galleries with visualizations of psychophysiological signals (in this case, EEG) may have reinforced empathy among our participants. This could also have influenced the reduction in psychological distance.

The intensity of user engagement may influence users’ perceived psychological distance to some extent. Higher user engagement in the ESVD group likely reduced the perceived psychological distance. Van Boven et al. (2010) showed that a reduction in objective distance and emotional intensity reduces perceived psychological distance. Emotional intensity also affects user engagement, as a good emotional design induces a sense of pleasure and security in the user, thus promoting user engagement during the experience (Lee et al., 2002).

### Social distance pre- and during COVID-19

Some studies describe psychological distance as the degree of divergence from the direct experience of the self, here and now, along with temporal, spatial, and social viewpoints or theoretical perspectives (Trope and Liberman, 2010). It is divided into dimensions such as temporal, spatial, and social viewpoints, or theoretical (Liberman and Trope, 2014). Scerrati et al. (2022) demonstrated that the pandemic likely drives the results of individual differences in the assessment of social proximity. Our study focuses on the “social distance” dimension, such as the distance between oneself and others.

Given that our participants were East Asians, we must discuss perceptions of social distance in these regions. In China, one regional aspect influencing social distance is *guanxi*, which is usually applied to business relationships, where the more the trust, the shorter is the social distance (Song et al., 2012). There is also an indication that social distance is slightly greater in urban settings (Ma et al., 2015). As for minorities, while the Chinese perceive some regional and international minorities as close, others are perceived far (Fong and Spickard, 1994).

Among minorities in Japan, indigenous people were considered as closer than people from mixed nationality backgrounds (Ball, 2009). In both countries, the social distance was greater when interacting with people with mental disabilities (Haraguchi et al., 2009; Ando et al., 2013), although physical contact was more frequent among Chinese nationals.

There was an indication of social isolation and depression among Japanese youth before the COVID-19 pandemic (Takagi et al., 2013), and such phenomena increased during the pandemic, including the suicide rate, particularly among women (Sugaya et al., 2020; Osaki et al., 2021). In China, poor mental health is associated with social distancing (Goodwin et al., 2021). The existing evidence suggests that such effects can be mitigated by family support (Li and Xu, 2022).

Social distancing is usually discussed in medical terms in the literature. In contrast, our study addresses social distancing as an opportunity for university students to experience art. Many of these students move out of their homes to pursue their education, and in the case of international students, they currently face the challenge of adapting to a different country amid a global pandemic. These factors can exacerbate isolation. Thus, we propose an alternative to decreasing the perception of social distance, with the potential mitigation of isolation among East Asian students, which could aid in addressing other mental and physical health issues.

**Figure 6:**
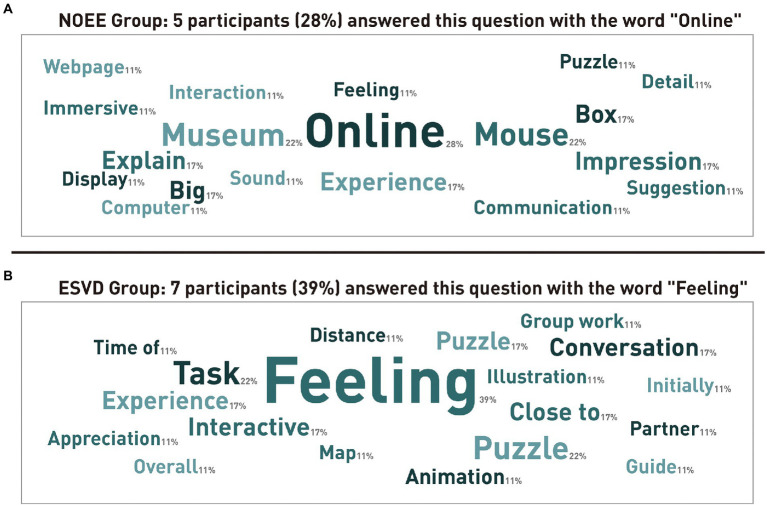
**(A)** High-frequency words that appeared in participants’ descriptions of the overall experience in the NOEE group (including percentages); **(B)** High-frequency words that appeared in participants’ descriptions of the overall experience in the ESVD group (including percentages).

We discovered in the feedback that the participants in the ESVD group were more concerned about their partners with whom they experienced the online exhibition together than those in the NOEE group. We also collected textual feedback from participants in the study. Figure 6 compares the keywords that appeared in the final feedback messages and the number of occurrences between the participants in the NOEE and ESVD groups. The percentage in the bottom-right corner of each keyword in the figure represents the percentage of participants who mentioned that keyword. The larger the value, the larger is the area shown in the figure. Overall, 36 of the 40 participants (18 in each of the two groups) submitted feedback. We found that the high-frequency word that was mentioned by five out of 18 participants and was the highest of all words in the NOEE group was “online” (28%); in the ESVD group, that was mentioned by seven out of 18 participants and was the highest of all words it was “feeling” (39%). The comparison between the two groups revealed that the words mentioned by the participants in the ESVD group were more often related to the partners who participated in the experiment, such as “Close to” (17%), “Conversation” (17%), “Group work” (11%), “Partner” (11%), “Distance” (11%), etc. (see Figure 6A). The words mentioned by the participants in the NOEE group were more related to online experiences, such as “Museum” (22%), “Mouse” (22%), “Experience” (17%), and “Impression” (17%), “Explain” (17%), etc. (see Figure 6B).

### Study limitations

Our study has several limitations. First, the sample size was relatively small. A more extensive sample size is necessary to provide more meaningful data to evaluate the efficacy of using an EEG signal visualization system for the user experience of an online exhibition. The results of this study might have differed if the sample included older adults or people from nonurban settings.

The second is the relatively single mode of experience. In this study, we used the GA&C website as a case study. The GA&C website is mainly experienced by users through computers, which are used more frequently than VR and AR devices in daily life for most users (Udell, 2019). With the evolution of virtual exhibitions, experience modalities have become more diverse. Using technologies such as Web/X3D, VR, and AR, visitors are offered the possibility of exploring virtual museums, interacting with virtual exhibits in real time, and visualizing these exhibits in contexts such as 3D gallery spaces (Petridis et al., 2005; Styliani et al., 2009). Additionally, the web-based form of online museums is one of the earlier developed and most popular ways to experience virtual museums; therefore, we chose this form as a way for participants to experience them in this study. Participants visited virtual museums in different modes of experience, which produced different effects. In the future, we will add to this study and continue to explore the impact of different experiential modes on participants’ psychological distance and engagement.

Additionally, we observed that participants did not significantly observe the state of EEG visualization images during the experience of focusing on the online exhibition, possibly because the online experience and EEG visualization were displayed on separate screens. In this case, the participants needed to raise their heads to move their eyes from the computer screen to the monitor. While the participants were fully engaged in viewing the artwork and reading information about it, their eyes were mainly focused on the computer screen. They spent less time observing the screen displaying the EEG signals. Therefore, if the EEG signal is displayed on the same screen as the artwork, participants are likely to spend more time focusing on the changes in the EEG signal, which may have a different effect on the results of the experiment.

## Conclusion

The COVID-19 pandemic has resulted in the need to maintain social distancing in public places; thus, the distance between people has been affected. Work (Hodder, 2020), study (Azorín, 2020; Bozkurt et al., 2020), exhibition viewing (Hoffman, 2020), etc., have been compulsorily shifted online to reduce the exposure of face-to-face contact. The distance between people and others has changed, becoming less perceptible and more complex. The development of remote services may change the way we perceive psychological distance. Our study reflects the “social distance” between people in post-pandemic situations. We also explore how the visualization of EEG signal differences in a virtual space can help reduce the psychological distance between users.

In this study, we developed and provided an EEG signal visualization system that provides a new way for users to experience online exhibitions and understand each other’s emotions. Users employing the system in different spaces can learn the differences in each other’s EEG signals through visualized images. We used printed pictures during the experiment to evoke the feeling of “psychological distance” between participants and others (Kundrát and Rojková, 2021). Within-group and between-group analyses were conducted for both groups by using independent sample *t*-tests and one-way ANOVA. User engagement was also investigated by using the UES-SF questionnaire, and repeated ANOVA was used to compare the differences between the two data groups. We summarize the following findings for both research questions:

- R.Q.1: Based on independent samples *t*-tests and one-way ANOVA analysis and on observations during the experimental procedure, we concluded that participants in the ESVD group perceived a significantly closer psychological distance between themselves and the participants on the opposite side than those in the NOEE group (*t* = −2.699; *p* = 0.008 < 0.05); additionally, participants experienced Task3 with significantly closer psychological distance assessments than that of Task1 (*p* = 0.002 < 0.05), Task2 (*p* = 0.000 < 0.05), and Task4 (*p* = 0.001 < 0.05).
- R.Q.2: Based on the repeated ANOVA analysis, we concluded that participants in the ESVD group had higher overall user engagement than the NOEE group, with marginal significance (*p* = 0.056 < 0.1).

In future research, we will continue to improve the presentation of EEG signal visualization. For example, we will combine the visualization with an online exhibition interface to make it easier for users to view and analyze whether the design of the visualization graphics affects the psychological distance between the users to some extent. Additionally, we intend to explore whether there are any similarities in the brain function network maps of two participants who are simultaneously experiencing the online museum; we also aim to determine whether the visualization of brainwaves affect the state of a user’s brain functional network map to a certain extent. We will continue to analyze the raw EEG data of the participants during the experience, whether there is a link between it and EEG visualization, and whether there is a link between it and the user-perceived mental distance. Regarding users’ physiological signals, we will continue to explore what other physiological signals (e.g., heartbeat, body temperature, and eye movements) can be visualized to help users better perceive psychological distance in addition to EEG signals. Additionally, in future experiments, we will increase the diversity of the sample (participants of different ages, nationalities, occupations, etc.), and the participants will not be limited to college students.

## Data availability statement

The raw data supporting the conclusions of this article will be made available by the authors, without undue reservation.

## Ethics statement

The studies involving human participants were reviewed and approved by the ethics review office of the Faculty of Library, Information and Media Science of the University of Tsukuba in Japan, the permission number is 22-4. The patients/participants provided their written informed consent to participate in this study. Written informed consent was obtained from the individual(s) for the publication of any potentially identifiable images or data included in this article.

## Author contributions

JL designed the study, conducted the experiments, performed the statistical analysis, analyzed and interpreted the data, created the images and tables, drafted the manuscript, and reviewed and revised the manuscript. YY participated in the design of the Moiré Pattern and the discussion of the EEG visualization scheme in the study. ZZ participated in the construction and implementation of the EEG visualization system. NY participated in the implementation of the experiments, assisted in the collection of data, took videos of the process, and reviewed and revised the manuscript. VX provided research guidance, wrote part of the discussion section, and critically reviewed and revised the manuscript. YO provided research guidance, research funding, experimental sites, laboratories, instrumentation, and other material resources related to the experiments. All authors contributed to the article and approved the submitted version.

## Funding

This research was supported by the Japan Science and Technology Agency, SPRING, Grant number JPMJSP2124 and Core Research for Evolutional Science and Technology, number JPMJCR1781.

## Conflict of interest

The authors declare that the research was conducted in the absence of any commercial or financial relationships that could be construed as a potential conflict of interest.

## Publisher’s note

All claims expressed in this article are solely those of the authors and do not necessarily represent those of their affiliated organizations, or those of the publisher, the editors and the reviewers. Any product that may be evaluated in this article, or claim that may be made by its manufacturer, is not guaranteed or endorsed by the publisher.
